# Thermoresponsive Alginate-Graft-pNIPAM/Methyl Cellulose 3D-Printed Scaffolds Promote Osteogenesis In Vitro

**DOI:** 10.3390/gels9120984

**Published:** 2023-12-15

**Authors:** Aikaterini Gialouri, Sofia Falia Saravanou, Konstantinos Loukelis, Maria Chatzinikolaidou, George Pasparakis, Nikolaos Bouropoulos

**Affiliations:** 1 Department of Materials Science, University of Patras, 26504 Patras, Greece; katerinagialoyri@gmail.com; 2 Department of Chemical Engineering, University of Patras, 26500 Patras, Greece; faliasaravanou@hotmail.com; 3 Department of Materials Science and Technology, University of Crete, 70013 Heraklion, Greece; loukelisk@yahoo.com; 4 Foundation for Research and Technology Hellas (FORTH), Institute of Electronic Structure and Laser (IESL), 70013 Heraklion, Greece; 5 Foundation for Research and Technology Hellas, Institute of Chemical Engineering and High Temperature Chemical Processes, 26504 Patras, Greece

**Keywords:** sodium alginate, PNIPAM, 3D printing, MC3T3-E1, bone tissue engineering

## Abstract

In this work, a sodium alginate-based copolymer grafted by thermoresponsive poly(*N*-isopropylacrylamide) (PNIPAM) chains was used as gelator (Alg-g-PNIPAM) in combination with methylcellulose (MC). It was found that the mechanical properties of the resulting gel could be enhanced by the addition of MC and calcium ions (Ca^2+^). The proposed network is formed via a dual crosslinking mechanism including ionic interactions among Ca^2+^ and carboxyl groups and secondary hydrophobic associations of PNIPAM chains. MC was found to further reinforce the dynamic moduli of the resulting gels (i.e., a storage modulus of ca. 1500 Pa at physiological body and post-printing temperature), rendering them suitable for 3D printing in biomedical applications. The polymer networks were stable and retained their printed fidelity with minimum erosion as low as 6% for up to seven days. Furthermore, adhered pre-osteoblastic cells on Alg-g-PNIPAM/MC printed scaffolds presented 80% viability compared to tissue culture polystyrene control, and more importantly, they promoted the osteogenic potential, as indicated by the increased alkaline phosphatase activity, calcium, and collagen production relative to the Alg-g-PNIPAM control scaffolds. Specifically, ALP activity and collagen secreted by cells were significantly enhanced in Alg-g-PNIPAM/MC scaffolds compared to the Alg-g-PNIPAM counterparts, demonstrating their potential in bone tissue engineering.

## 1. Introduction

Tissue engineering is a growing interdisciplinary field in biomedical sciences combining materials science, chemistry, biology, medicine, and engineering sciences. The basic aim of tissue engineering is to develop biological substitutes that maintain, restore, or improve tissue function [1]. Today, a diverse range of biomaterials of synthetic or biological origin are widely used in clinical practice for tissue engineering applications. Examples of synthetic biomaterials include metals, polymers, ceramics, and composite materials. Commonplace examples of natural biomaterials are protein or polysaccharide-based biomaterials such as collagen, gelatin, chitosan, and silk. In addition, autologous grafts or decellularized biomaterials are widely used for tissue regeneration [2]. One of the limitations of using the classical techniques to fabricate tissue engineering scaffolds is the resulting poor microstructural architecture and the restriction needed to control interconnections between the pores. Compared to traditional techniques, three-dimensional (3D) printing makes reproducible and customized structures with near-perfect micro-architecture and morphology [3]. Several 3D constructs with applications in regenerative medicine have been fabricated in recent years. The most common printing techniques used are laser based, jet and extrusion based printing, and fused deposition modeling [4].

Of particular interest are printing methods that allow for the direct deposition of viscous aqueous mixtures in arbitrary shapes, such as soft scaffolds that support the proliferation of mammalian cell populations and tissue growth. In this context, responsive polymers have been utilized to construct 3D matrices that can respond to external stimuli and exert shape/volume change in a reversible and dynamic manner and often recapitulate intrinsic properties of the extracellular matrix (ECM). Arguably, temperature-responsive polymers are widely studied due to their versatile responsive properties near physiological body temperature, as well as their ability to tune their stimuli response fully isothermally by combination with other co-monomers and/or polymers [5]. In addition, responsive polymer networks can undergo large changes in their dynamic moduli, rendering them ideal soft biomaterials for 3D printing in biomedical applications [6].

Poly(*N*-isopropylacrylamide) (PNIPAM) has been studied as a thermoresponsive polymer because of its rapid phase transition, biocompatibility and lower critical solution temperature (LCST) at approximately 32 °C, which is close to physiological body temperature. PNIPAM contains both hydrophilic amide groups (–CONH–) and hydrophobic isopropyl (–CH(CH_3_)_2_) side chains; in an aqueous environment, PNIPAM chains undergo a reversible sol-gel transition. Below the LCST, the chains are fully dissolved in water, and the polymer exhibits coil-like conformation due to hydrogen bonding and van der Waals forces. Above the LCST, the chains become hydrophobic, leading to a globule-like structure [7,8,9].

The printability and the biological evaluation of many thermosensitive hydrogels of synthetic or biological origin have been reported in the literature. For instance, bioengineered 3D-printed skin constructs based on thermosensitive PNIPAM hydrogels have been successfully evaluated for skin tissue engineering [10]. Pluronic F-127, a polaxamer co-polymer composed of polyethylene oxide (PEO) and polypropylene oxide (PPO), was used in combination with gelatin and hyaluronan to fabricate vascular channels [11]. Furthermore, the new synthetic biocompatible polymer PolyIsoCyanide was 3D-printed in a complex hydrogel construct and used as a fugitive material that could be removed after thermal stimulation [12]. Moreover, incorporation of particles into thermosensitive hydrogels has been reported to create hybrid hydrogels with improved rheological and mechanical properties, which in turn improve their printability [13].

Alginate is a major polysaccharide found in marine brown seaweed. Sodium alginate is a biopolymer broadly used in the food and beverage, pharmaceutical, cosmetics and medical industries, and has attracted significant interest due to its biocompatibility and biodegradability. It can be modified via covalent bonding of functional compounds, due to the abundant carboxylate and hydroxyl units, leading to new properties and applications in wound healing, controlled delivery of bioactive molecules, and cell encapsulation. The most common method to crosslink sodium alginate and form a hydrogel is the use of divalent cations such as calcium ions (Ca^2+^), resulting in crosslinks ionically formed by the “egg-box” model [14].

Methylcellulose (MC) is derived from cellulose, a linear polysaccharide comprising glucose units held together by 1-4-β-glucosidic linkages, the most abundant renewable polymer in nature synthesized from plants, algae, fungi, and some bacterial species [15]. Cellulose is biocompatible and has significant mechanical strength; however, natural cellulose is insoluble in water, limiting its biomedical applications. To overcome this, the hydroxyl group of cellulose can be substituted with a methyl group. MC is a water-soluble biopolymer utilized in pharmaceutics, cosmetics and food industry as an emulsifier or as a thickening agent. In hydrogel 3D-printing processes, MC improves rheological properties by enhancing ink viscosity, which is necessary to produce high-quality printed structures. In addition, several studies utilize MC-based hydrogels for cell engineering applications, emphasizing the in vitro biocompatibility of this polysaccharide [16].

The scope of the present work is to investigate the 3D printability of synthetic Alg-g-PNIPAM and Alg-g-PNIPAM/MC hydrogels. To this end, the thermoresponsive behavior of the corresponding polymers was studied, and the printing profile of the hydrogels was characterized by rheological analysis. Furthermore, the structural and morphological characterization of the 3D-printed scaffolds were investigated, while their in vitro erosion characteristics were evaluated. The cytocompatibility of the fabricated scaffolds was assessed in terms of cell viability, proliferation, adhesion, and morphology using the pre-osteoblastic cell line MC3T3-E1, whose osteogenic behavior has been previously reported as tunable in the presence of thermoresponsive polymers [17]. Alkaline phosphatase (ALP) activity, calcium, and total collagen production by cells cultured on scaffolds were evaluated as osteogenic markers to validate the potential of the developed scaffolds in bone tissue engineering applications.

## 2. Results and Discussion

### 2.1. Synthesis of Alg-g-PNIPAM

**Figure 1:**
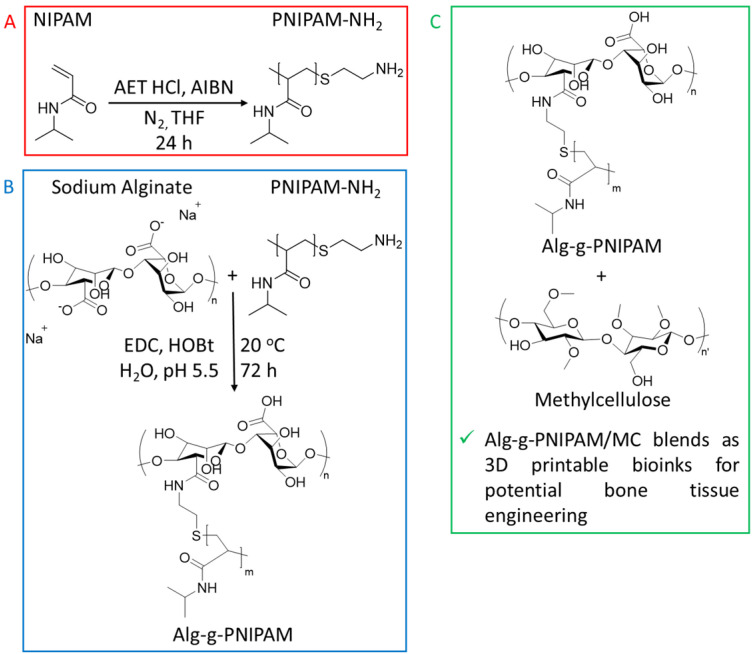
Synthetic route of the sodium alginate-based graft copolymer (**A**,**B**) and its combination with MC for the final ink material (**C**).

The proposed Alg-g-PNIPAM bioink was synthesized via free radical polymerization of the amino-terminated PNIPAM chains followed by a grafting procedure on the sodium alginate backbone by carbodiimide chemistry (Figure 1). The formation of the final Alg-g-PNIPAM product was confirmed by ^1^H NMR and FTIR characterization. The peaks at 3.5–4.6 ppm correspond to the four protons of the alginate ring [18]; the six methyl protons of the isopropyl group of NIPAM are depicted at ~1.1 ppm, the methylene and methine protons at 1.3–2.2 ppm, and the proton of the isopropyl group are linked to the amide group (N-C-H) at ~3.9 ppm (Figure S1). From the FTIR data, the 3305 cm^−1^ band is representative of the aminoterminated groups of the PNIPAM-NH_2_ precursor, which are absent in the spectrum of the Alg-g-PNIPAM due to their conversion to amide bonds (dashed area, Figure S2). Additionally, the characteristic amide C=O stretching and N-H bending of the PNIPAM are observed in the FTIR spectra of both the PNIPAM-NH_2_ polymer and the Alg-g-PNIPAM at 1750–1500 cm^−1^, further indicating the successful grafting reaction (Figure S2).

### 2.2. Thermosensitivity Measurements and Rheological Evaluation

**Figure 2:**
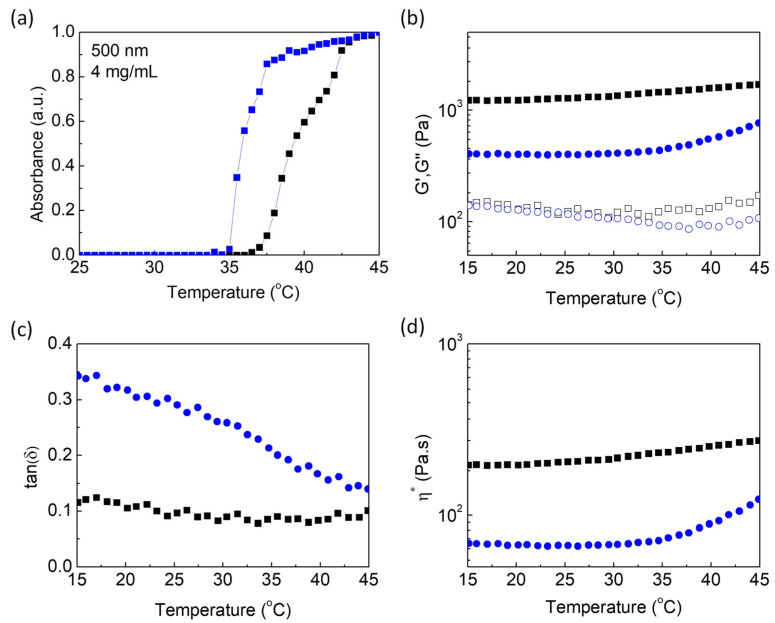
(**a**) Lower critical solution temperature measurements of PNIPAM (blue) and Alg-g-PNIPAM (black) polymers; (**b**) G′ (closed) and G″ (open) moduli, (**c**) tan (δ), and (**d**) complex viscosity as a function of temperature of Alg-g-PNIPAM (blue, circles) and of Alg-g-PNIPAM/MC (black, squares) aqueous solutions at a frequency of 6.28 rad/s, a strain amplitude of 0.1%, and during the heating cycle with a heating rate of 1 °C/min.

The thermoresponsive behavior of PNIPAM precursor and Alg-g-PNIPAM was evaluated at a concentration of 4 mg/mL by measuring the absorption at 500 nm at different temperatures, i.e., 25–45 °C. The value of LCST was determined as the temperature onset point at which the solution turned cloudy. Both polymers exhibit sharp thermosensitive behavior. A LCST value at 35 °C was measured for PNIPAM slightly above 32 °C, which is attributed to the hydrophilic amino-end moieties that are known to shift the LCST at higher temperatures [19]. In the presence of alginate, the respective value for Alg-g-PNIPAM is again shifted at 37 °C, as presented in Figure 2a. This result is expected due to the hydrophilic nature of the alginate backbone, and interestingly, it is close to physiological body temperature, implying that the graft copolymer can be a good candidate for biomedical applications.

Alg-g-PNIPAM and Alg-g-PNIPAM/MC were used as main gelators to deconvolute the effect of each individual macromolecular component. The formulations were dissolved in 2 mM Ca^2+^ aqueous solution. Oscillatory shear experiments reveal a thermo-induced gelation of the systems due to the hydrophobic associations of the PNIPAM pendants. Notably, the gel strengthening is observed at T > 35 °C (the LCST of the PNIPAM-NH_2_ chains) upon a heating cycle at a ramp rate of 1 °C/min and a constant frequency of 6.28 rad/s; the storage modulus (G′) rapidly surpassed the loss one (G″), and the tangent δ is less than 0.2 for both systems. The rheological behavior of the 3D networks is enhanced in the entire temperature region, i.e., G′ constantly exceeds G″ by additional ionic interactions between the negatively ionized carboxylic groups of alginate or hydroxyl groups of MC and the positively charged Ca^2+^ divalent cations, according to the “egg-box” mechanism, as presented in Figure 2b,c. The proposed f factor, i.e., f = [Ca^2+^]/[COO^−^] or f = [Ca^2+^]/([COO^−^] + [OH^−^]) in molar ratio, is equal to f = 0.0095 in the Alg-g-PNIPAM hydrogel and f = 0.0056 in the Alg-g-PNIPAM /MC sample. Furthermore, the MC-thickening component creates a stable hydrogel adequate for 3D extrusion biomedical applications, as the G′ of the network is ~1280 Pa at room temperature and is equal to ~1500 Pa at physiological body temperature, i.e., a ~68% increase compared to the storage modulus of the MC-free network, while the tan (δ) of the MC-enriched hydrogels remains at ~0.1.

Moreover, in Figure 2d, the temperature dependance of the complex viscosity is demonstrated. The examined thermal region could be divided in two sections below and above ca. 35 °C. At T < 35 °C, the viscosity of the hydrogels remains constant as the 3D networks are formed due to ionic interactions, whereas at T > 35 °C, the secondary hydrophobic associations due to the PNIPAM pendant chains increase the viscosity values of the gelators. Indicatively, the viscous expansion between room and body temperature is 16% for the Alg-g-PNIPAM/MC system and 15% for the Alg-g-PNIPAM gel.

**Figure 3:**
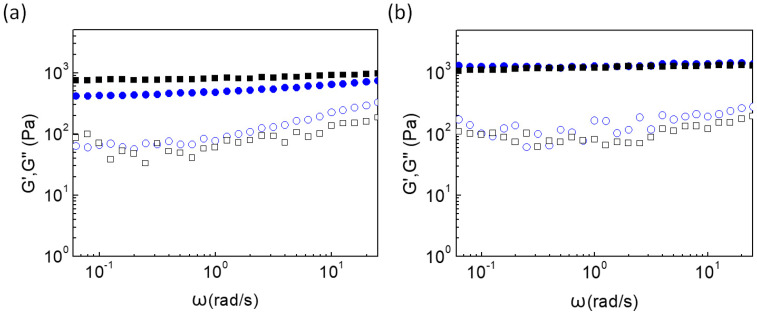
G′ (closed), G″ (open) versus angular frequency at (**a**) 25 °C and (**b**) 40 °C of Alg-g-PNIPAM (blue, circles) and of Alg-g-PNIPAM/MC (black, squares) hydrogels.

Additionally, the dependence of the storage and loss moduli was studied as a function of the angular frequency at room temperature (Figure 3a) close to the printer-bed temperature, i.e., 40 °C (Figure 3b). The G′ and G″ moduli of both gelators are almost independent of the frequency changes, denoting a solid-like behavior at both examined temperatures. At low temperature, the ionic interactions predominate in the network formation, whereas the MC augments the stability of the gel. Upon heating, the coexistence of the ionic and hydrophobic associations results in stronger hydrogel matrices.

**Figure 4:**
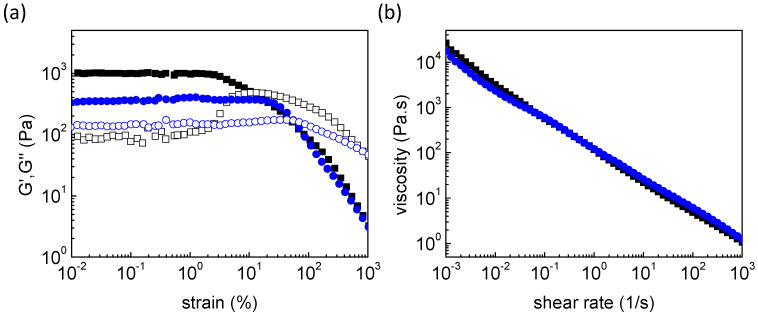
(**a**) G′ (closed) and G″ (open) upon strain sweep test and (**b**) viscosity as a function of shear rate at 25 °C (printing temperature) of Alg-g-PNIPAM (blue circles) and of Alg-g-PNIPAM/MC (black squares) hydrogels.

Considering the proposed materials as potential candidates for soft 3D printing, strain sweep and shear rate sweep tests were conducted in contemplation of bio-printability through extrusion. At low strain amplitude, the MC-rich system presents a stronger-gel behavior compared to the MC-free sample. As the strain amplitude is increased, the liquid-like threshold of the Alg-g-PNIPAM/MC is at a strain amplitude of approximately 10%, and the yield point of the Alg-g-PNIPAM is at 55%, denoting an easier-to-print material, as seen in Figure 4a. Besides, the shear-thinning effect is an important design criterion for efficient and accurate 3D printability, and both gelators meet this requirement, as shown in Figure 4b.

### 2.3. Characterization and Erosion Studies of 3D-Printed Scaffolds

#### 2.3.1. Structural Characterization

**Figure 5:**
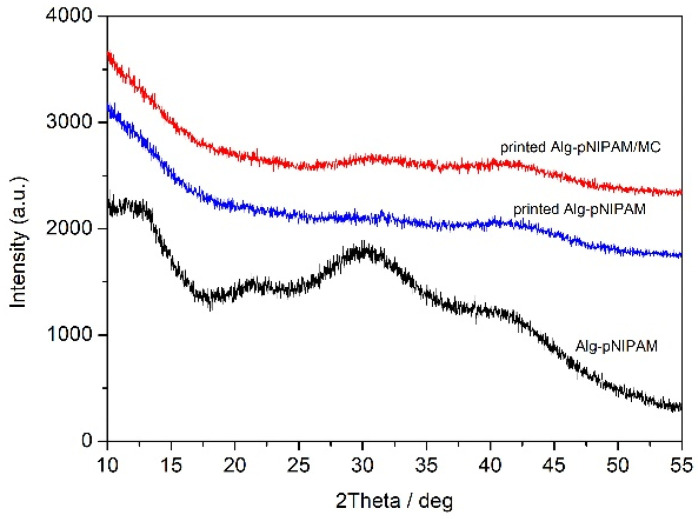
XRD patterns of Alg-g-PNIPAM and 3D-printed structures of Alg-g-PNIPAM and Alg-g-PNIPAM/MC.

Figure 5 shows the diffraction patterns of the dry hydrogel before printing and the lyophilized 3D-printed structures. All the diffractograms exhibit broad peaks, indicating the amorphous structure of all materials.

#### 2.3.2. Thermal Characterization

**Figure 6:**
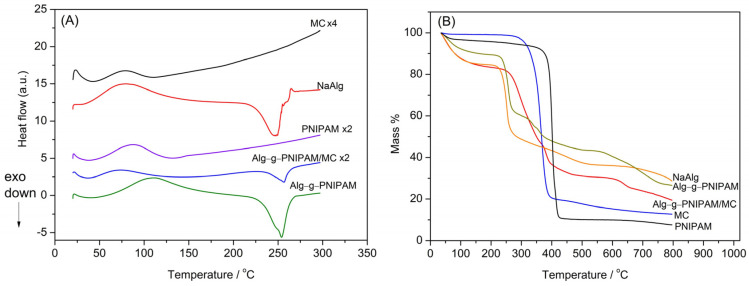
DSC (**A**) and TGA thermograms (**B**) of raw materials and printed objects. The DSC graphs have been shifted and multiplied by a factor for clarity.

Thermogravimetric analysis was used to study the thermal behavior of raw materials and the printed samples. The results are depicted in Figure 6.

DSC thermogram of MC shows an endothermic peak at 77 °C, due to the loss of water (Figure 6A). The thermal behavior of sodium alginate is characterized by an endothermic peak at 79 °C, also attributed to water loss. A broad endothermic peak at 247 °C corresponds to degradation and depolymerization [20]. In the case of PNIPAM, except the water loss endotherm peak at 87 °C, the glass transition temperature Tg is observed at 148 °C, while the polymer is thermally stable at least until 300 °C [21]. Comparative assessment of both hydrogel inks Alg-g-PNIPAM and Alg-g-PNIPAM/MC revealed similar thermal behavior. Both samples are characterized by an alginate degradation peak at 253 and 257 °C, respectively.

In the TGA thermogram of MC, a decomposition peak is observed, which starts at 259 °C and finishes at 412 °C with a mass loss of 80%, while the total mass loss at 800 °C is 87% (Figure 6B). The sodium alginate decomposition occurs in three steps. The first step from 35 °C to 196 °C is due to dehydration, while decomposition starts at 196 °C and ends at 500 °C. Finally, the weight loss from 500 °C to 800 °C is attributed to Na_2_CO_3_ formation [20]. The thermogram of PNIPAM displays one distinctive degradation step which starts at 311 °C, due to degradation of the backbone of the polymer, and ends at 428 °C. Weight loss is continued until 800 °C due to main chain degradation [22,23]. Comparing the thermograms of sodium alginate with the grafted Alg-g-PNIPAM, it is shown that the grafted polymer exhibits higher thermal stability at least until 700 °C. After blending the Alg-g-PNIPAM copolymer with MC, the thermogram is more complex, and at 800 °C, the two copolymers Alg-g-PNIPAM and Alg-g-PNIPAM/MC show a remaining mass of 27 and 19%, respectively. The different thermal behavior of Alg-g-PNIPAM/MC can be attributed to the lower thermal stability of MC in comparison with sodium alginate and PNIPAM.

#### 2.3.3. Morphological Characterization

**Figure 7:**
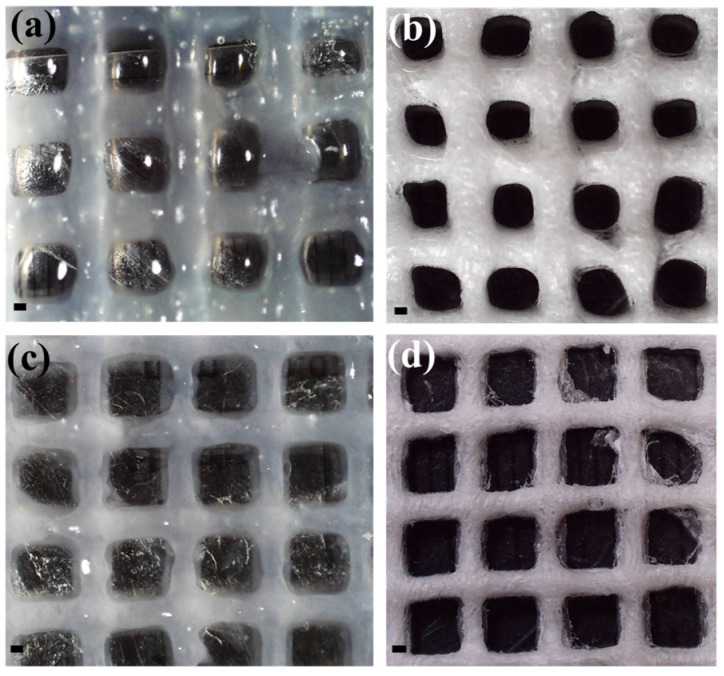
Digital microscope images of Alg-g-PNIPAM (**a**) before and (**b**) after freeze-drying and Alg-g-PNIPAM/MC (**c**) before and (**d**) after freeze-drying. Scale bar is equal to 500 μm.

The mean pore size of the wet Alg-g-PNIPAM sample was found equal to 2.04 ± 0.18 mm, and the dry 1.37 ± 0.14 mm, respectively. On the other hand, the average pore size in the presence of MC is 1.97 ± 0.17 mm for the wet scaffolds and 1.80 ± 0.11 mm for the dry ones. The examination of scaffolds’ geometry revealed a decrease in pore size after freeze-drying, for both compositions, as seen in Figure 7.

**Figure 8:**
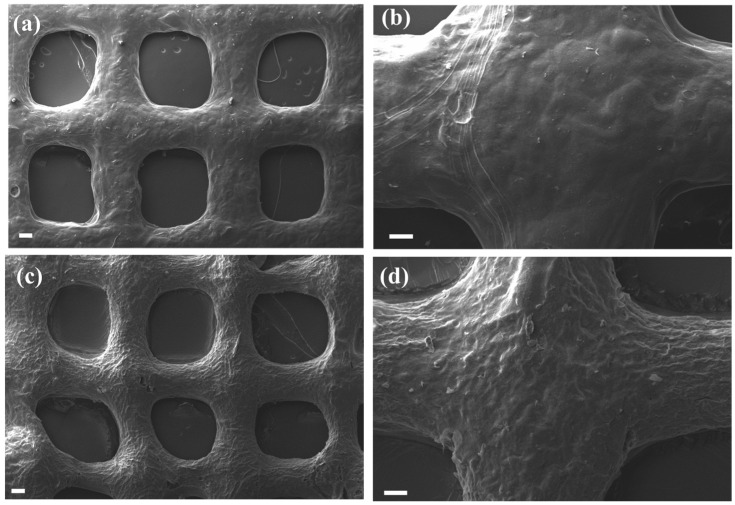
SEM images of 3D-printed scaffolds of Alg-g-PNIPAM (**a**,**b**) and Alg-g-PNIPAM/MC (**c**,**d**). Scale bar for (**a**,**c**) represents 200 μm and (**b**,**d**) is equal to 100 μm.

Scanning electron microscopy (SEM) images from the lyophilized scaffolds are shown in Figure 8. Both compositions maintain their porous structure after lyophilization. It is interesting to mention that both samples show a rounded pore geometry, which is expected based on the soft nature of the gels. At a higher magnification, it can be observed that the specimen containing MC has a rougher surface texture.

#### 2.3.4. Erosion Studies

**Figure 9:**
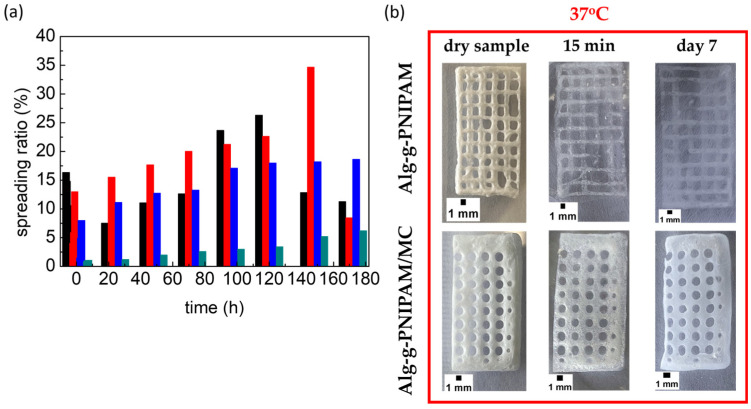
(**a**) Spreading ratio (%) defined as the percentage change in the pattern line width compared to the dry structure of Alg-g-PNIPAM at 20 °C (black) and at 37 °C (red) and of Alg-g-PNIPAM /MC at 20 °C (blue) and at 37 °C (cyan); (**b**) Photographs of the swelling samples up to day 7 at 37 °C.

The erosion profile of 3D-printed Alg-g-PNIPAM/MC and Alg-g-PNIPAM domains have been examined, and the results are presented in Figure 9. The 3D-printed designs were freeze-dried, followed by hydration with distilled water at room and body temperature. The more stable material, Alg-g-PNIPAM/MC, was degraded only by 6% at 37 °C and 19% at 20 °C after 7 days (172 h). In contrast, Alg-g-PNIPAM was eroded by up to 26% at 20 °C after 5 days (120 h) and by up to 35% at 37 °C after 6 days (144 h). After these timeframes, the weaker bonds of Alg-g-PNIPAM domains were disintegrated. In Figure 7b, the moisturized MC-enriched and the MC-free samples are illustrated at day 7. After a week, the Alg-g-PNIPAM/MC seems almost intact compared to the Alg-g-PNIPAM. Hence, it was concluded that the MC-rich scaffolds could be promising candidates for a two-week pre-osteoblastic cell culture investigating cell viability, proliferation, and osteogenic differentiation, considering that MC is expected to enhance the cell proliferation.

### 2.4. Evaluation of Cytocompatibility, Cell Adhesion, Viability and Proliferation

#### 2.4.1. Cell Viability and Proliferation

**Figure 10:**
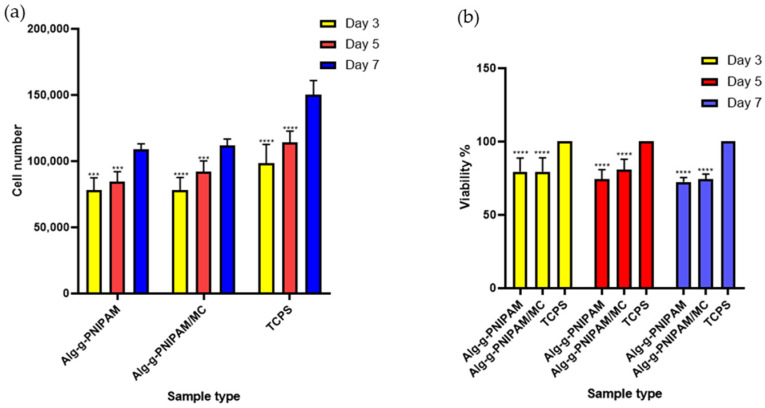
Cell viability and proliferation of pre-osteoblastic cells seeded on Alg-g-PNIPAM, Alg-g-PNIPAM/MC scaffolds and TCPS control at 3, 5 and 7 days expressed as OD values (**a**) and as cell viability percentage (**b**). Bars represent averages ± standard deviation of *n* = 6 (*** *p* < 0.001, **** *p* < 0.0001).

Cell viability and proliferation using pre-osteoblastic cells have been assessed at days 3, 5, and 7 (Figure 10a,b). The number of cells increased from day 3 to day 5 and up to day 7, demonstrating that the scaffold compositions promote cell proliferation. Both scaffold compositions, Alg-g-PNIPAM and Alg-g-PNIPAM/MC, showed similar cell viability at each time point, and these were found to be significantly lower compared to the tissue culture polystyrene (TCPS) control; however, they reached 80% viability of the control. The Alg-g-PNIPAM/MC scaffolds indicated a higher cell viability on day 5; however, this was not significant compared to the Alg-g-PNIPAM counterparts. These results show that the Alg-g-PNIPAM scaffolds are cytocompatible. Statistical analysis of each scaffold composition compared to the TCPS control revealed significant differences (*p* < 0.0001) at all time points. Another report on Alg-g-P(NIPAM)-based solutions of various concentrations showed 80% fibroblast cell viability after 24 h, indicating the cytocompatibility of this grafted co-polymer [20].

#### 2.4.2. Cell Adhesion and Morphology Evaluation

**Figure 11:**
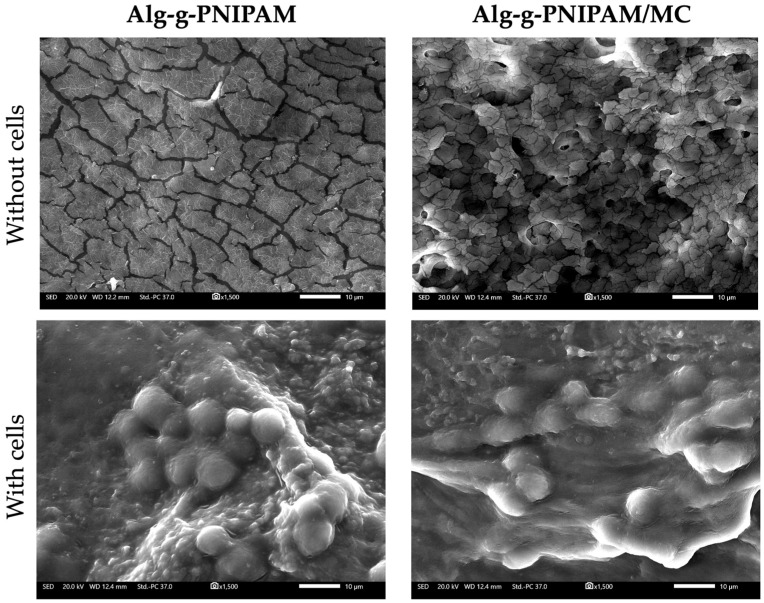
SEM images of scaffolds without (**upper panel**) and with cells (**lower panel**) at day 7. The surface of the Alg-g-PNIPAM scaffold is depicted in the upper left and Alg-g-PNIPAM/MC in the upper right images. The elongated morphology of adhered cells with visible cell nuclei is shown on the Alg-g-PNIPAM (**lower left**) and Alg-g-PNIPAM/MC (**lower right**) scaffolds. Magnification is ×1500, and scale bars represents 10 μm.

We observed the cell adhesion on the surface of the scaffold struts and their morphology by means of SEM. Figure 11 (upper panel) shows the pre-osteoblastic cells adhered on both scaffold compositions after 7 days in culture. The cell nuclei of a dense cell layer covering both scaffold types are clearly visible in Figure 11 (lower panel). The morphology of adhered cells did not show any differences between the two compositions. The characteristic morphology of cell nuclei indicates that both scaffold types support cell adhesion. Although none of the scaffold compounds, alginate [24], PNIPAM [25] or MC [15], possess cell-specific binding sites to promote cell adhesion, both scaffold compositions showed adequate attachment of pre-osteoblasts. Similarly, other studies report on good cell adhesion on alginate [26] and PNIPAM [27]-based scaffolds combined with other biomaterials or coatings.

#### 2.4.3. Evaluation of Osteogenic Differentiation Markers ALP Activity and Calcium and Collagen Production by Cells Cultured on Alg-g-PNIPAM and Alg-g-PNIPAM/MC Scaffolds

**Figure 12:**
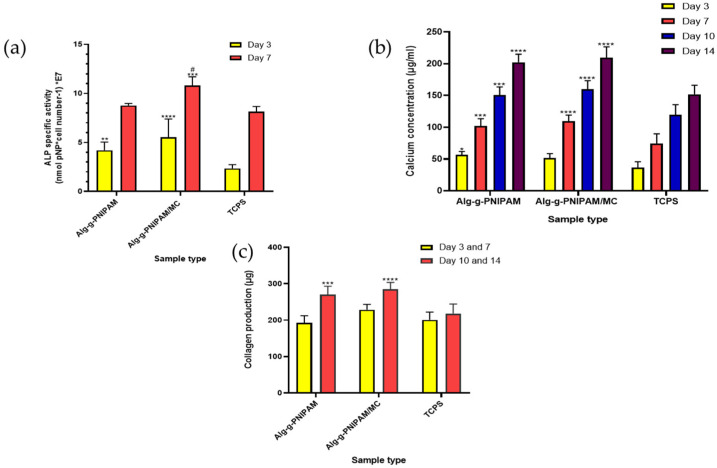
Normalized alkaline phosphatase activity of pre-osteoblasts cultured onto the two scaffold types and TCPS control on days 3 and 7 (**a**). Calcium concentration on days 3, 7, 10 and 14 (**b**). Collagen production by cells on days 7 and 14 (**c**). Bars represent averages ± standard deviation of *n* = 6 (** *p* = 0.0098, *** *p* = 0.0004, **** *p* < 0.0001). Asterisks (*) denote significant differences between each scaffold composition with the TCPS control, while hashtag (#) designates significant differences between Alg-g-PNIPAM and Alg-g-PNIPAM/MC scaffolds.

Bone formation is accompanied by specific enzymatic activity and expression characteristic of the osteoinduction process. Alkaline phosphatase (ALP) activity is elevated in areas of extracellular matrix mineralization. ALP activity was investigated at two early time points of culture, as it presents an early-phase osteogenesis marker. At day 3, both scaffold compositions demonstrate with a two-fold increase in enzyme activity, significantly higher than that of the TCPS control. At day 7, the ALP activity increased two-fold compared to day 3, and it is higher on both scaffold compositions compared to the control, with a significantly higher difference on the Alg-g-PNIPAM/MC scaffolds (Figure 12a).

The concentration of the calcium produced by osteoblasts was assessed on days 3, 7, 10 and 14 (Figure 12b) as a late marker of osteogenesis. The calcium concentration increased between the consecutive experimental time points. In particular, the production of calcium showed at least a 30% increase, with significantly higher levels in both scaffold types compared to the TCPS control at days 7, 10 and 14, with an approximate increase of 30–50% between the experimental timepoints. On days 7 and 10, the MC-containing scaffolds presented a significantly higher increase compared to the Alg-g-PNIPAM ones. Of note, the higher calcium content in both scaffold types may be attributed to the presence and release of Ca^2+^ used for crosslinking the hydrogels before and after 3D printing.

Osteoblasts produce more collagen type I than any other cell. We have quantified the total collagen amount produced by cells cultured onto the scaffolds on days 7 and 14 (Figure 12c). Rich collagen synthesis with a concentration of 750 μg/mL was measured on day 7 for Alg-g-PNIPAM and 900 μg/mL for Alg-g-PNIPAM/MC scaffolds, while secreted collagen concentration increased by 10–25% on day 14. Both scaffold compositions depicted comparable collagen production with the TCPS control at day 7. However, on day 14, both scaffold compositions showed significantly higher collagen levels compared to the TCPS control, with the Alg-g-PNIPAM/MC scaffolds displaying significantly higher levels compared to the Alg-g-PNIPAM scaffolds.

Alginate is one of the most prominently used biomaterials in bone tissue engineering due to its excellent gelling capacity and its ability to physically bind cations such as calcium and strontium, which are then available for release in aquatic environments, thus providing an osteogenic effect [28]. PNIPAM grafted onto gelatin has also been used as an injectable hydrogel for bone defect regeneration [29], making the mixing of these two biomaterials a rather promising combination for bone tissue engineering applications. The additional incorporation of MC into the alginate/PNIPAM blend was considered with regard to supporting the 3D-printing process, as well as due to its established osteogenic potential [30,31]. Alginate scaffolds have been previously reported to exhibit increased alkaline phosphatase activity, presenting elevated values between subsequent time points, compliant with the findings of this study [32]. Similarly, an injectable, thermo-responsive hyaluronic acid-g-chitosan-g-PNIPAM copolymer has been reported to show increased ALP activity and calcium deposition with progressing time in culture of bone marrow-derived mesenchymal stem cells [33]. In that particular work, calcium ions’ concentration was determined through alizarin red staining over a period of 21 days, displaying a gradual increase, thus indicating a similar pattern to that evident in our measurements of calcium concentration in the scaffold supernatants.

## 3. Conclusions

In this study, we showed that it is possible to combine graft thermoresponsive alginates with MC to produce soft inks suitable for 3D printing in biomedical applications. It was possible to control the dynamic moduli of the resulting gels by the interplay of the different stimuli such as temperature and Ca^2+^ ions. The presence of MC significantly improved the printing fidelity and the erosion profiles of the polymer networks post-printing. Cells cultured on both scaffold compositions, Alg-g-PNIPAM and Alg-g-PNIPAM/MC, displayed a significant increase in osteogenic markers including ALP activity, calcium, and collagen production compared to the control. Particularly, the MC-containing scaffolds indicated higher responses in these markers compared to the Alg-g-PNIPAM counterparts. These findings demonstrate the osteogenic potential of these gels and their excellent ability to act as 3D-printed scaffolds for bone tissue engineering.

## 4. Materials and Methods

### 4.1. Materials

*N*-isopropylacrylamide (NIPAM, Fluorochem), 2,2′-Azobis(2-methylpropionitrile) (AIBN, Sigma Aldrich, St. Louis, MO, USA), 2-aminoethanethiol hydro-chloride (AET HCl, Alfa Aesar), 1-hydroxybenzotriazole hydrate (HoBT, Fluka), 1-ethyl-3-(3-(dimethylamino) propyl) carbodiimide (EDC, Alfa Aesar), tetrahydrofuran (THF, Sigma Aldrich), calcium chloride dihydrate (CaCl_2_·2H_2_O, Sigma Aldrich), sodium hydroxide (NaOH, Panreac), and acetone (Sigma-Aldrich) were used as purchased. Purified water (3D-H_2_O) was provided by an ELGA Medica-R7/15 device. Sodium alginate (NaALG, Aldrich) with a molecular weight range of 120,000–190,000 g/mol and a mannuronic/guluronic ratio (M/G) of 1.53 (values are given by the supplier) was dissolved at 7 *w*/*v*% in 3D water, and was further purified against dialysis membrane (MWCO 12,000–14,000 Da) before being freeze-dried.

### 4.2. Synthesis of the Amino-Terminated PNIPAM-NH_2_ Side

The polymeric side chains were synthesized by free radical polymerization. AIBN was used as an initiator and AET HCl as a chain transfer agent. Briefly, 4 g (0.035 mol) of NIPAM monomer units were dissolved in 40 mL THF. The mixture solution was degassed with nitrogen for 15 min. Then, the mixture was heated at 70 °C. Then, 0.02 g (0.177 mmol, 0.5% over the monomer concentration) AET and 0.058 g (0.354 mmol, 1% over the monomer concentration) AIBN were added to the mixture. The mixture was left under stirring for 24 h. The reaction was stopped by exposure to air at room temperature followed by purification against distilled water with a dialysis membrane (MWCO: 12,000–14,000 Da), in order to remove unwanted byproducts and impurities; finally, the product was freeze-dried and stored as white flakes. The number average molecular weight (Mn) was evaluated by acid–base titration of the amino-terminated groups of the polymer chains, as presented in Table 1.

### 4.3. Synthesis of the Alg-g-PNIPAM

The grafting reaction of the thermosensitive PNIPAM-NH_2_ side chains onto the Alg backbone was accomplished by carbodiimide chemistry, forming an amide group by the carboxyl groups of the Alg monomer units’ ring and the -NH_2_ end-group of the PNIPAM chains [34,35]. EDC and HOBt were used as coupling agents. Then, 1.15 g of NaALG and 2.25 g of PNIPAM-NH2 were dissolved separately in 25 mL and 45 mL of 3D H_2_O, respectively. The mixtures were left under stirring in room temperature for 24 h. Then, the PNIPAM solution was added in the Alg one. Subsequently, 0.111 g (0.89 mmol, 5% moles over the monomer moles) HOBt dissolved in 3 mL of 3D-water and 0.6279 g (0.003 mol, 20% moles over the monomer moles) of EDC dissolved in 5 mL of 3D H_2_O were added to the mixture and left under stirring at 20 °C for 48h. The pH of the Alg/PNIPAM mixture was adjusted at 5–6, and the EDC solution was added in two steps (half of the amount was added at the start of the reaction, and the rest after 24 h). The final mixture product was precipitated in acetone in order to remove the un-grafted PNIPAM-NH_2_ chains, followed by filtration and dissolution of the precipitate in ~5 wt.% in distilled water. The pH of the mixture was set at 11 using NaOH (1M), and was further purified using a dialysis membrane (MWCO: 25,000 Da), before finally being freeze-dried. In Table 2 the molecular characteristics of the produced biopolymer are presented.

### 4.4. Proton Nuclear Magnetic Resonance (^1^H NMR)

The molar composition (%) of the grafting PNIPAM chains onto the Alg was calculated by integrating the above characteristic areas, and the weight composition (%) of the graft copolymer was calculated by the molar composition using the M_w_ building units of sodium alginate and the M_w_ of the PNIPAM side chains. The samples were dissolved in D_2_O (peak ~4.8 ppm) and were analyzed using a Bruker Avance iii Hd Prodigy Ascend Tm 600 MHz spectrometer (Billerica, MA, USA).

### 4.5. Hydrogel Preparation

A solution of 5 wt.% Alg-g-PNIPAM containing 2 mM of Ca^2+^ was prepared. Methylcellulose was added up to final mixture concentration 7.5 wt.%. The hydrogels were left under stirring at T = 10 °C up to full homogeneity.

### 4.6. Rheological Studies

The rheological evaluation of the Alg-g-PNIPAM and Alg-g-PNIPAM/MC hydrogel samples was conducted on a stress-controlled AR-2000ex rheometer (TA Instruments) with a cone and plate geometry (diameter 20 mm, angle 3°, truncation 111 μm). The hydrogels were loaded on a Peltier plate, which highly ensures the experimental temperature (±0.1 °C), and their thermo- and shear-responsiveness were measured. A solvent trap was located over the cone–plate geometry to prevent changes in the hydrogels’ concentrations. All the measurements were operated in the linear viscoelastic regime (LVR), which has been verified for its sample by a strain sweep test and a constant angular frequency at 6.28 rad/s.

### 4.7. Determination of the LCST

The LCST temperature of PNIPAM and NaAlg-PNIPAM was measured by using a Shimadzu UV-1900i spectrophotometer (Shimadzu Co., Kyoto, Japan) at a wavelength of 500 nm. The instrument was equipped with a Peltier-controlled thermostated cell holder (TCC-100, Shimadzu Co., Kyoto, Japan)). Measurements were performed in the two polymer solutions at a concentration of 4 mg/mL.

### 4.8. 3D Scaffold Design and Manufacturing

The pattern of the 3D model designed using the online TinkerCAD™ design platform and exported to an .stl file. By loading the digital design on the open-source slicing software Ultimaker Cura 5.2.1, all printing parameters were set, and the final file exported in .gcode format. The generated gcode file was loaded to the 3D printer. The scaffolds were manufactured with a low-cost 3D FFF (fused filament fabrication) printer, which was modified to a hydrogel printer. In summary, the extruder of a Wanhao Duplicator i3 plus was removed, and next, a syringe support was designed and manufactured using a polylactic acid filament in a commercial 3D printer (Wanhao Duplicator i3 plus, Wanhao Ltd., Jinhua, China). A plastic 10 mL syringe was inserted in the extruder’s position, and the head of the syringe was connected with a clamp to a pneumatic dispenser (DX-250, Metcal, Hampshire, UK). At the edge of the syringe, a plastic nozzle with a diameter of 400 μm was connected to a Luer lock fitting. The bed temperature was set at 40 °C, and a 60 mm glass Petri dish was adhered on the printing bed by means of tape. Afterwards, the hydrogel was placed in the syringe. An external air compressor (Mini 50, Airblock, Thessaloniki, Greece) was joined to the syringe, and by applying the appropriate pressure, the syringe plunger was forced to extrude the hydrogel through the nozzle. The 3D-printed objects were crosslinked with calcium ions directly by immersion after printing in a 0.5 M CaCl_2_·2H_2_O solution for 30 min. Finally, the scaffolds were washed thoroughly with distilled water and lyophilized (Telstar Cryodos, Terrassa, Spain) [30].

### 4.9. Structural Characterization

X-ray diffraction patterns were collected with a Bruker D8 Advance diffractometer (Bruker AXS GmbH, Karlsruhe, Germany) with monochromatic CuKα1 radiation (λ = 1.5406 nm) at a voltage of 40 kV and a current value of 40 mA. The angular scanning speed was 0.35 s/step. After lyophilization, the dried gels were placed on an XRD specimen holder and compacted with a glass slide to flatten them.

ATR-FTIR spectroscopy was performed on an FTIR spectrometer (IR Tracer-100, Schimadzu, Kyoto, Japan) using the ATR accessory MIRacle™ Single Reflection equipped with a ZnSe crystal. After the background signal collection, a small amount of the dried sample was placed in contact with the ATR crystal, and a pressure of 75 psi was applied. Spectra were recorded by averaging 25 scans at a resolution of 4 cm^−1^ in the spectral range between 550 and 4000 cm^−1^.

### 4.10. Thermal Characterization

For the differential scanning calorimetry (DSC) measurements, powdered samples (1–3 mg) were weighed and placed in aluminum pans, sealed hermetically, and assessed in a Q200 (TA Instruments, New Castle, DE, USA) differential scanning calorimeter. An empty aluminum pan used as the reference sample. The specimens were heated from 25 to 300 °C at a heating rate of 10 °C/min under nitrogen atmosphere at a flow rate of 50 mL/min.

Thermogravimetric analysis (TGA) measurements were performed through a TG Q500 thermogravimetric analyzer (TA Instruments, New Castle, DE, USA). About 5 mg of the samples were placed in a platinum pan and heated over a temperature range from 35 to 800 °C at a heating rate of 10 °C/min, under nitrogen with a flow rate of 60 mL/min.

### 4.11. Morphological Characterization

Scanning electron microscopy images (Zeiss EVO MA-10, Carl Zeiss, Oberkochen, Germany) determine the surface morphology of the 3D-printed scaffolds. The samples were placed on aluminum holders, fixed with a conductive silver paste, and then gold sputtered (BAL-TEC SCD-004). Also, images were taken using a digital microscope (Celestron LLC, Torrance, CA, USA) to measure the differences in pore diameters before and after freeze-drying. Pore sizes were quantified using ImageJ analysis software (Version 1.44p, National Institutes of Health: Bethesda, MD, USA).

### 4.12. Cell Culture Maintenance

MC3T3-E1 pre-osteoblastic cells (DSMZ Braunschweig, Germany) derived from mouse calvaria were cultured in a humidified incubator at 37 °C and 5% CO_2_ (ThermoFisher, Waltham, MA, USA) in alpha-MEM supplemented with 10% (*v*/*v*) fetal bovine serum (FBS), 100 g/mL penicillin and streptomycin, 2 mM l-glutamine and 2.5 μg/mL amphotericin (all from PAN-Biotech, Aidenbach, Germany) (complete alpha-MEM). Cells were cultured to 90% confluence by medium change every two days, and detached using trypsin-0.25% ethylenediaminetetraacetic acid (EDTA) (Gibco, Thermo Fisher Scientific, Waltham, MA, USA) for passaging. All experiments were conducted with cell passages from 10 to 12. For the osteogenic potential assessment of the scaffolds, we applied osteogenic medium comprising complete alpha-MEM supplemented with 10 nM dexamethasone, 10 mM glycerophosphate, and 50 g/mL l-ascorbic acid 2-phosphate (Sigma-Aldrich, St. Louis, MO, USA).

Prior to cell seeding, all scaffolds were sterilized by immersion in 70% ethanol for 3 min, followed by 30 min of UV irradiation at 265 nm at both sides. Each scaffold was submerged for 10 min in culture medium. Then, the medium was removed and a 15 μL cell suspension with 3 × 10^4^ cells was seeded onto each scaffold in a 96-well plate; 150 μL cell culture medium was added, and cell-loaded scaffolds were placed in the incubator.

### 4.13. Cell Viability and Proliferation Assessment

Cell viability assessment of cells cultured onto 3D-printed scaffolds is essential in any tissue engineering application. The cell viability of the scaffolds was investigated by employing a metabolic assay, the PrestoBlue™ (Invitrogen Life Technologies, Carlsbad, CA, USA) cell viability assay. The reagent contains resazurin, which changes color from blue to purple according to cell metabolism. On each scaffold, 10 μL of PrestoBlue™ reagent and 90 mL of fresh medium were added to a final volume of 100 mL for each well of a 96-well plate. The mixture was incubated for 1 h at 37 °C and then transferred to another 96-well plate to measure the absorbance in a spectrophotometer at 570 and 600 nm (Synergy HTX Multi-Mode Micro-plate Reader, BioTek, Bad Friedrichshall, Germany). The number of living cells was measured photometrically at 3, 5, and 7 days. For the determination of cell numbers from the absorbance values, we used a calibration curve of known pre-osteoblastic cell numbers in the same multi-well plate type.

### 4.14. Cell Adhesion and Morphology

The cell adhesion and morphology onto the scaffolds were monitored using SEM at 7 days. Prior to microscopy, the scaffolds were rinsed with phosphate-buffered saline (PBS) to remove the remaining culture medium, and were fixed using 4% paraformaldehyde for 20 min. The scaffolds were then dehydrated in increasing ethanol concentrations from 30, to 100% *v*/*v*. The scaffolds were then sputter-coated with a 20 nm gold layer (Baltec SCD 050, Baltec, Los Angeles, CA, USA) and observed by means of a scanning electron microscope (JEOL JSM-6390 LV, Tokyo, Japan) at an accelerating voltage of 20 kV.

### 4.15. Osteogenic Potential Evaluation of Pre-Osteoblasts Seeded onto Scaffolds by Determination of the ALP Activity, Collagen and Calcium Secretion

The ALP activity is indicative of the initial stages of osteogenesis. Briefly, the cell-seeded scaffolds remained in culture for 3 and 7 days using osteogenic medium. Each scaffold was rinsed with PBS and submerged in 200 μL lysis buffer (0.1% Triton X-100 in 50 mM Tris-HCl pH 10.5) to extract the cell lysate. The mixture of 100 μL lysate with 100 μL of 2 mg/mL p-nitrophenyl phosphate (pNPP, Sigma, St. Louis, MO, USA) solution was incubated at 37 °C for 1 h, and measured photometrically at 405 nm. The enzymatic activity was calculated using the following equation:

Units = mmol p-nitrophenol/min

and normalized to total protein in lysates determined using the Bradford protein concentration assay.

Calcium is one of the elemental components of bone tissue. Calcium mineralization is a crucial regulator for the formation of the bone matrix and a late marker of osteogenesis. Calcium secretion was determined by the O-cresol phthalein complexone (CPC) method. Culture supernatants were collected after 3, 7, 10 and 14 days, and 10 μL of each sample was mixed with 100 μL of calcium buffer and 100 μL of calcium dye CPC. The final solutions were transferred to a 96-well plate to measure the absorbance at 405 nm.

Collagen is a crucial element and the primary organic component of bone tissue, which plays a pivotal role in providing structural support in the formation of the extracellular matrix (ECM). Quantification of collagen secretion in culture supernatants was performed by the Sirius Red (Direct red 80, Sigma-Aldrich, St. Louis, MO, USA) staining method after 7 and 14 days. In brief, 25 μL of supernatants were diluted in 75 μL of ultrapure water at each time point. The solution was mixed with 1 mL of 0.1% Direct Red 80 and incubated at room temperature. After centrifugation of the samples, the pellets were rinsed with 0.5 M acetic acid for non-bound dye removal. Finally, 200 μL of NaOH 0.5 M was added to extract the collagen-bound dye complex. The absorbance of the solutions was measured with a spectrometer in a 96-well plate at 530 nm. A calibration curve correlates the quantity of collagen to mg/mL.

Statistical analysis was performed using GraphPad Prism 8.0.2 software (GraphPad Software, San Diego, CA, USA) and a two-way ANOVA followed by Tukey’s multiple comparisons test. All values are expressed as average ± standard deviation (SD). The adjusted p value set * *p* < 0.05 compared each scaffold composition with the TCPS control at each time point. Statistical analysis was performed to compare the two scaffold compositions, Alg-g-PNIPAM and Alg-g-PNIPAM/MC.

## Figures and Tables

**Table 1:** Molecular characteristics of the PNIPAM-NH_2_.

Grafting Chain	Mn (g/mol) ^a^
PNIPAM-NH_2_	13,740

^a^ From acid–base titration.

**Table 2:** Molecular characteristics of the polymer.

Graft Copolymer	M_w_ (g/mol) ^a^	% Molar Composition Alg/Grafting Chain (mol/mol) ^b^	% Weight Composition Alg/Grafting Chains (*w*/*w*)	Number of PNIPAM Side Chains Per NaALG Backbone ^b^
Alg-g-PNIPAM	167,470	74.4/25.6	83.6/16.4	2

^a^ Calculated by the following equation: M_w,cop_ = M_w,Alg_/wt% Alg, M_w,Alg_ = 140,000 g/mol; ^b^ Calculated by ^1^H NMR.

## Data Availability

All data and materials are available on request from the corresponding author. The data are not publicly available due to ongoing research using a part of the data.

## Supplementary Materials

The following supporting information can be downloaded at: https://www.mdpi.com/article/10.3390/gels9120984/s1, Figure S1. ^1^H-NMR spectra of (a) Alg-g-PNIPAM and of (b) PNIPAM-NH_2_. Figure S2. FTIR spectra of Alg-g-PNIPAM (a), Alg (b), and PNIPAM-NH2 (c).
