# Supplementary material for: Thermoresponsive Alginate-Graft-pNIPAM/Methyl Cellulose 3D-Printed Scaffolds Promote Osteogenesis In Vitro
Source: Gels. 2023 Dec 15;9(12):984. doi: 10.3390/gels9120984 (PMC10743144; doi:10.3390/gels9120984)
Supplement: Supplementary file 1 [file gels-09-00984-s001.zip › gels-2739615-supplementary.pdf]

Supplementary Materials

Thermoresponsive Alginate-Graft-pNIPAM/Methyl Cellulose 3D-Printed Scaffolds Promote Osteogenesis In Vitro

Aikaterini Gialouri [1,†], Sofia Falia Saravanou [2,†], Konstantinos Loukelis [3], Maria Chatzinikolaidou [3,4,*], George Pasparakis [2,*] and Nikolaos Bouropoulos [1,5,*]

^{1}H NMR

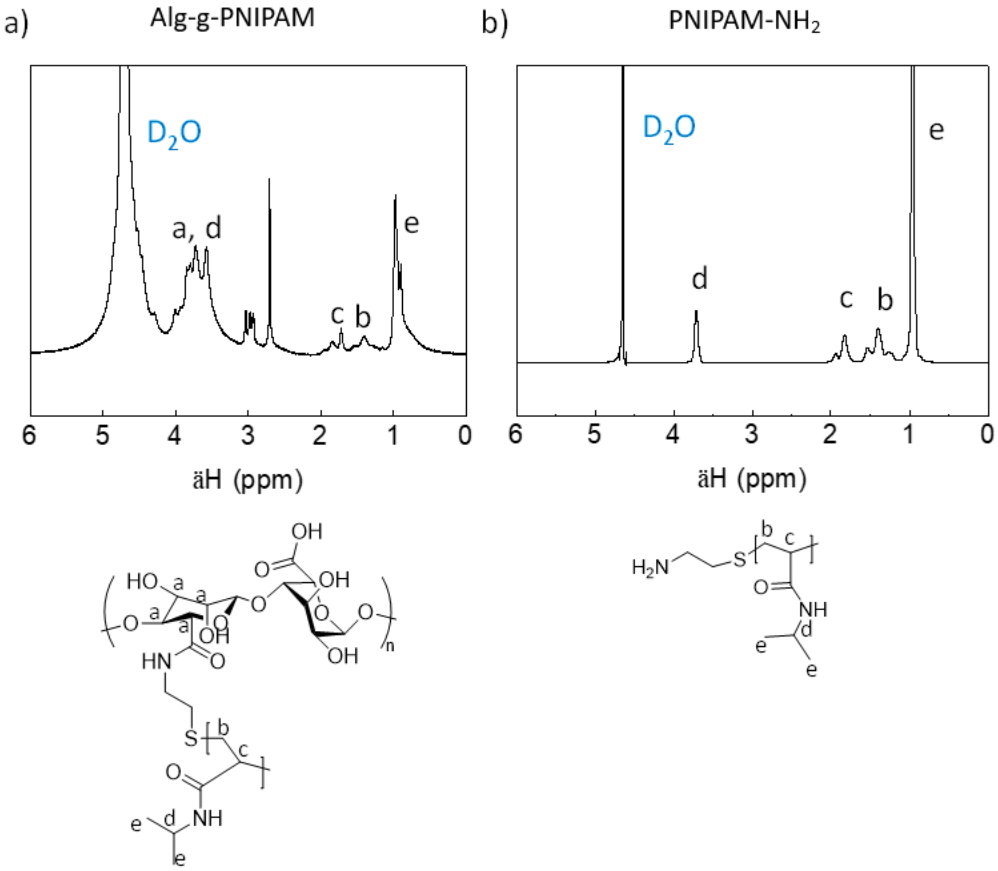

Figure S1. ^{1}H-NMR spectra of a) Alg-g-PNIPAM and of b) PNIPAM-NH_2.

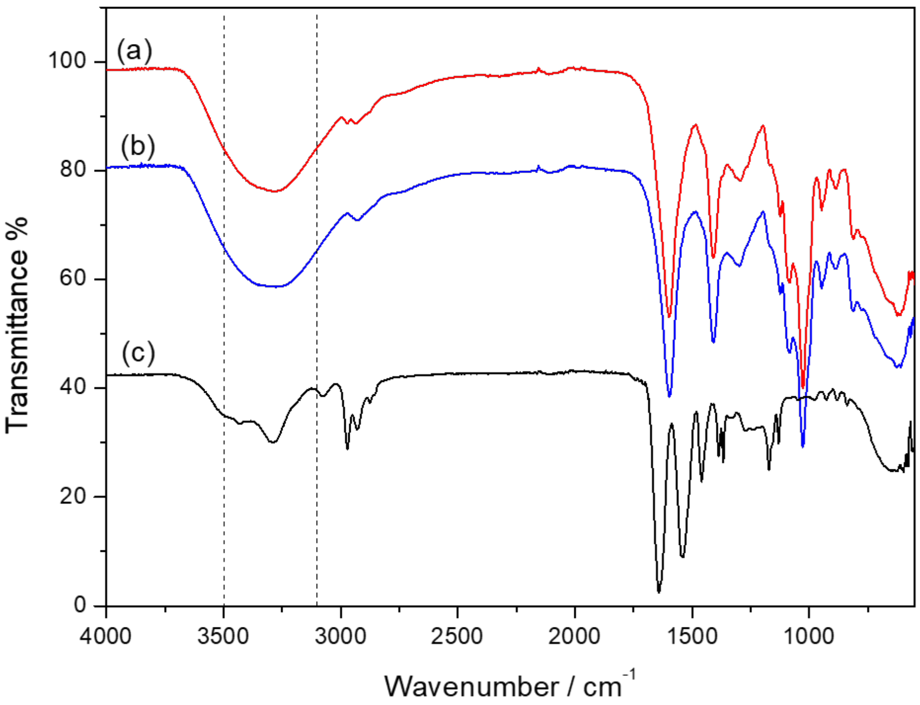

Figure S2. FTIR spectra of Alg-g-PNIPAM (a), Alg (b), and PNIPAM-NH_2 (c).
